# Lifestyle, caries, and apical periodontitis: Results from a university‐based cross‐sectional study

**DOI:** 10.1111/iej.14165

**Published:** 2024-11-12

**Authors:** Carlo Gaeta, Giulia Malvicini, Dominga Di Lascio, Marco Martignoni, Gabriele Ragucci, Simone Grandini, Crystal Marruganti

**Affiliations:** ^1^ Unit of Endodontics and Restorative Dentistry, Department of Medical Biotechnologies University of Siena Siena Italy; ^2^ Private Practice in Milan Milan Italy

**Keywords:** apical periodontitis, caries, lifestyle, Mediterranean diet, physical activity, stress

## Abstract

**Aim:**

Lifestyle factors significantly influence the development of inflammatory diseases, and emerging evidence suggests they may also impact oral health. However, no studies have explored their role in apical periodontitis (AP) amongst adults. This study aimed to assess the association between adherence to the Mediterranean diet (MD), physical activity, perceived stress, and sleep quality with the periapical and caries status in a university‐based cohort.

**Methodology:**

A total of 149 periodontally healthy patients were included in the study. Clinical assessments and radiographic examinations [Orthopantomography (opt) and periapical radiographs] were conducted to evaluate the periapical status. Data on their periapical index (PAI) score and the decayed, missing and filled teeth (DMFT) index were recorded. Validated questionnaires were used to investigate patient's lifestyles. A final logistic regression model was performed for the multivariable analysis to evaluate the predictive ability of adherence to Mediterranean lifestyle on the presence of AP; other local, systemic and environmental factors were included as independent factors in the model.

**Results:**

Significant associations were observed between AP and high/low adherence to the MD (*p* = .00), high/low‐moderate physical activity (*p* = .00), high/low sleep quality (*p* = .00) and high/low perceived stress (*p* = .00). The final multivariable regression model showed that low adherence to MD (OR = 3.68; 95% confidence interval [CI]: 1.24–10.83; *p* = . 01) and reduced sleep quality (OR = 3.04; 95% CI: 1.42–6.50; *p* = .00) were identified as potential risk factors for AP development. On the other hand, the DMFT index showed no significant association with lifestyle factors (OR = 1.0; CI: 1.01–1.14; *p* = .02) but was correlated with the development of AP (OR = 1.07; CI: 1.01–1.14; *p* = .02).

**Conclusion:**

Results from the present study suggest a potential association between low adherence to MD and reduced sleep quality with the development of AP. Individuals with low adherence to MD and inadequate sleep quality faced respectively 4‐fold and 3‐fold increased odds of developing periapical lesions. Further research is essential to elucidate the causal mechanisms underlying these associations and to determine whether lifestyle adjustments could improve endodontic success rate.

## INTRODUCTION

Unhealthy lifestyle behaviours significantly contribute to the global burden of noncommunicable diseases (NCDs), which are the leading cause of disability and account for approximately 63% of all fatalities (Kushner & Sorensen, 2013; Marrero et al., 2012). These adverse behaviours encompass inadequate nutrition, unhealthy body weight, lack of physical activity, poor sleep quality, stress and substance abuse, such as tobacco and alcohol (Kushner & Sorensen, 2013). In recent years, there has been a growing interest in unravelling the potential benefits of adopting healthy lifestyle factors in reducing the risk of major chronic diseases (Parkinson et al., 2023; Zhang et al., 2021).

Individuals who embrace healthy lifestyle behaviours, characterized by adherence to healthy dietary principles (often referred to as strong adherence to the Mediterranean diet (MD)), regular physical activity and nonsmoking habits, have been associated with a reduced risk of developing NCDs, cardiovascular diseases (CVD), as demonstrated in various studies (Chiuve et al., 2011; Stringhini et al., 2010).

Mounting evidence demonstrates that modifiable risk factors such as lifestyle habits may play a role in the onset of oral diseases (Li et al., 2024). Recent studies support the link between lifestyle behaviours and periodontitis, with a higher prevalence and increased severity observed in individuals with unhealthy lifestyle habits compared to those with healthier behaviours (Marruganti et al., 2022, 2023). A recently published study conducted on a cohort of paediatric patients has highlighted the presence of a possible correlation between high adherence to the MD and a decreased likelihood of having decayed deciduous teeth (Franciosi et al., 2024). Furthermore, a prior study involving young males showed that the percentage of participants with healthy dentition rose consistently as physical activity levels increased (Huttunen et al., 2023). High levels of physical fitness were found to be a protective factor against the need for additional dental restorative treatments (Huttunen et al., 2023).

The negative impact of unhealthy lifestyles on overall health is often attributed to the onset of low‐grade systemic inflammation (LGSI) and the overproduction of reactive oxygen species, leading to oxidative stress (Esposito et al., 2004; Frodermann et al., 2019). Apical periodontitis (AP) is characterized as a chronic inflammatory condition with a microbial origin (Siqueira Jr & Rôças, 2022; Tibúrcio‐Machado et al., 2021). There is compelling evidence indicating that AP contributes to LGSI, with existing literature suggesting that AP enhances systemic inflammation by elevating proinflammatory markers such as cytokines, chemokines, C‐reactive protein, interleukin 6, asymmetric dimethylarginine and C3 levels (Braz‐Silva et al., 2018; Georgiou et al., 2019; Gomes et al., 2013).

A recent systematic review indicates that approximately 52% of the global adult population is affected by at least one tooth with AP (Tibúrcio‐Machado et al., 2021). Despite the recognized significance of lifestyle factors in the development and progression of various inflammatory diseases, there is currently no available literature regarding the association between unhealthy lifestyle behaviours and the subsequent occurrence of periapical diseases.

Therefore, the aim of the current cross‐sectional study was to investigate the relationship between patients' lifestyles and the presence of AP, the extent of periapical bone destruction (PAI) and their decayed, missing and filled teeth (DMFT) index.

## MATERIALS AND METHODS

### Study design

The present study is reported according to the Strengthening the Reporting of Observational Studies in Epidemiology (STROBE) guidelines for cross‐sectional studies (von Elm et al., 2008). The research protocol was approved by the local ethics committee of the Region of Tuscany South‐Eastern Area (protocol number: 18993/2021) and was registered on Clinicaltrial.gov (NCT06072742).

### Setting and participants

All consecutive patients attending the Unit of Endodontics at the University of Siena were screened between September 2020 and August 2021. Patients were eligible based on the following inclusion criteria:

- Age between 18 and 70 years old.
- Ability and willingness to give written consent.
- Presence of at least six teeth.

The exclusion criteria were:

- Age < 18 years.
- Pregnancy or lactation.
- Periodontitis (Page & Eke, 2007).
- Nonendodontic lesions in maxilla/mandible (Chauhan et al., 2019).
- Administration of antibiotics within the last 6 months.
- Inability and unwillingness to give written consent.
- Patients diagnosed with AP on teeth with inadequate endodontic treatments and coronal restorations (Ng et al., 2011).

Participants were enrolled in the study after they read and signed the written informed consent in accordance with the Declaration of Helsinki.

### Variables

#### Socio‐demographic characteristics

Patient‐related data, including patients' age, gender, smoking and oral hygiene habits, occupation and education level, were recorded. Furthermore, self‐reported information regarding comorbidities that could affect the susceptibility to AP was registered. These included Inflammatory bowel disease (IBD), rheumatoid arthritis (RA), diabetes and osteoporosis. The medical reports were checked for verification in case the patients self‐reported to be affected by one of the mentioned comorbidities.

#### Lifestyle behaviours

Lifestyle behaviours were assessed by administering a set of four validated questionnaires: (i) adherence to the Mediterranean Diet questionnaire (QueMD) (Gnagnarella et al., 2018); (ii) International Physical Activity Questionnaire (IPAQ) (Mannocci et al., 2010); (iii) Italian version of the Perceived Stress Score (IPSS‐10) questionnaire (Cohen et al., 1983) and (iv) Pittsburgh Sleep Quality Index (PSQI) questionnaire (Curcio et al., 2013; Mondo et al., 2021). According to the sum scores obtained in each questionnaire, participants were categorized as having high versus low adherence to MD, moderate/high versus low physical activity level, low versus moderate/high perceived stress and good versus poor sleep quality. All the questionnaires were independently administered by two examiners (C.G. and D.D.) blinded to the periapical status of the patients, who posed structured questions and provided explanations. To obtain consistency and appropriateness between the two examiners, a thorough calibration process was conducted prior to data collection. During this process, the examiners reviewed the validated questionnaires together to align their understanding of each question's intent and relevance. They standardized the procedures for administering the questions, discussing best practices to handle any potential variations in responses. Additionally, the examiners performed practice interviews, which allowed them to refine their approach and ensure that the questions were posed consistently across all participants.

Furthermore, smoking was self‐reported and assessed categorically (yes/no). Alcohol consumption was measured using the recommended intake as a threshold (one standard alcoholic drink a day for women and two standard alcoholic drinks a day for men) and participants were divided into groups based on whether they were drinking more or less than the recommended amount (Ricci et al., 2020).

#### Dietary assessment

Two examiners (C.G. and D.D.), administered a validated 15‐item questionnaire called QueMD to assess how well patients followed the MD (Gnagnarella et al., 2018). This questionnaire covered foods commonly associated with MD and other frequently consumed items. Participants selected their consumption frequency from five options for each component, with portions standardized for the Italian population. An alternate MD score (aMed) was calculated based on QueMD responses, assigning 1 point for each food consumption above Italian national levels (National Research Institute for Food and Nutrition, 2003) for each MD item: 1–2 glasses of wine per day for men and 1 glass per day for women, red meat (≤1–3/week), fish (≥2/week), dried fruits (≥2/week), pulses (≥2/day), wholegrain goods (≥1/day), veggies (≥2/day), fresh fruits (≥2/day) and olive oil (≥3/day) (Bach‐Faig et al., 2011; Gnagnarella et al., 2018). The sum of aMed score ranged from 0 (minimal MD adherence) to 9 (maximal MD adherence). It was categorized as low (aMed <5) or high (aMed >4) adherence using the median in this study's population as the threshold (Marruganti et al., 2022).

#### Perceived stress assessment

The clinical examiners administered the Italian version of a validated 10‐item questionnaire designed to assess patients' perceived stress levels (IPSS‐10) (Cohen et al., 1983). The questionnaire was composed of 10 questions with response options ranging from 0 (never) to 4 (very often). Most questions were negatively stated (scored from 4 to 0), except for four positively stated questions (scored from 0 to 4; items 4, 5, 7 and 8). Scores were totaled after reversing the positive items' scores and ranged from 0 to 40, with higher scores indicating higher perceived stress levels. Participants were categorized based on the IPSS‐10 score, with a classification of moderate/high stress (IPSS‐10 >13) or low perceived stress (IPSS‐10 ≤13) (Marruganti et al., 2023) following established guidelines (Biswas et al., 2019, State of New Hampshire Employee Assistance Program, 1983).

#### Physical activity assessment

Physical activity level was evaluated using the validated short version of IPAQ (Mannocci et al., 2010). The questionnaire comprised seven items that inquired about the frequency and duration of intense and moderate physical activity and walking or sedentary activities in the past week. The weekly activity times were computed based on the number of days and minutes spent engaging in vigorous, moderate and walking/light activities. The total score was expressed in metabolic equivalents (METs) per week and it was determined by multiplying the weekly activity time by the intensity‐specific metabolic values, following the instructions provided in the IPAQ scoring guidelines. The total physical activity level was categorized as low, moderate or high using the IPAQ automatic report (https://theipaq/home). The IPAQ automatically categorized the overall physical activity level as low, moderate or high based on the responses (Marruganti et al., 2022).

#### Sleep quality assessment

Sleep quality was evaluated using the validated Italian version of the PSQI questionnaire (Curcio et al., 2013; Mondo et al., 2021). The questionnaire comprised seven domains (subjective sleep quality, sleep latency, sleep duration, habitual sleep efficiency, sleep disturbances, use of sleeping medications and daytime dysfunction), each rated on a scale from 0 to 3. The total scores varied from 0 to 21, with higher scores indicating poorer sleep quality. Participants with a total score of 5 or above were categorized as experiencing ‘poor sleep quality’, whilst those with lower scores were classified as having ‘good sleep quality’ (Marruganti et al., 2023).

#### Clinical examination

All patients underwent extra and intra‐oral examinations. The periapical status was investigated by palpation, percussion, thermal cold testing and panoramic radiographs. Afterwards, teeth that exhibited deep carious lesions, deep restorations, no response to pulp testing or painful response to biting and/or percussion or palpation were suspected of AP (American Association of Endodontists, 2013). Those teeth underwent further periapical radiograph using the long cone paralleling technique with a film holder (Duncan et al., 2023) and a beam‐aiming radiographic unit (Nomad Pro 2; KavoKerr, Biberach, Germany) operating at 60 kV and 7 mA. Photostimulable phosphor plates (VistaScan [Dürr Dental Beitigheim‐Bissinger, Germany]) were used as receptors, and the exposure time for the radiographs varied, with a range of 0.2 ms for anterior teeth and 0.25–0.32 ms for posterior teeth. For image evaluation, a dedicated display was used to visualize digital radiographic images (Vistascan, Dürr Denta, Beitigheim‐Bissinger, Germany).

The following data were recorded (Malvicini et al., 2024):

- DMFT index (World Health Organization, 2013).
- Presence of AP.
- Periapical Index Score (PAI) to assess periapical status (Ørstavik et al., 1986).
- Periodontal status using a periodontogram (Papapanou et al., 2018).
- Identification of lesions unrelated to endodontic causes in the maxilla and mandible (Chauhan et al., 2019).

Furthermore, the quality of root canal treatment and coronal restoration of endodontically treated teeth was evaluated. Indeed, the quality of the root canal treatments and the coronal restorations were judged by the two (D.D. and M.G.) examiners following the criteria described by Ng et al. (2011). Precisely, the quality of the previous treatment was considered satisfactory if a well‐compacted root filling extended to within 2 mm of the radiographic root apex (Ng et al., 2011). If the quality of either the root canal treatment or the coronal seal was not within the standard, the entire treatment was considered inadequate. Only those patients in which AP was diagnosed on teeth with adequate endodontic treatments and coronal restorations were considered eligible for inclusion.

#### Periapical index

Periapical health was assessed radiographically using the PAI score (Ørstavik et al., 1986) which was determined through visual inspection of the periapical area, assigning a numerical value based on the extent and severity of inflammation. Scores ranged from 0 to 5, as follows:

1. Normal periapical structures.
2. Minor changes in bone structure.
3. Changes in bone structure with slight mineral loss.
4. Periodontitis with well‐defined bone circumscription and a halo of bone sclerosis.
5. Severe periodontitis with significant bone loss and a diffuse radiolucent image.

Scores 1 and 2 indicated periapical health, whilst scores 3, 4 and 5 indicated AP. The choice of the assigned score adhered to established guidelines (Ørstavik et al., 1986). PAI score was assessed by two different observers (D.D. and G.M.). The two observers underwent a calibration process involving reviewing 100 standard radiographs previously scored by the index developers. Any discrepancies in their evaluations were resolved through discussion. This calibration process was repeated twice within 2 weeks, and inter‐ and intra‐observer agreements were measured using kappa values. After calibration, both observers independently reviewed periapical radiographs of the teeth under standardized conditions. For multirooted teeth, the highest PAI score amongst individual roots was considered. In case of uncertainty, a consensus was reached, and the higher scores were selected. Notably, during the assessment, the observers were unaware of the patients' identities and clinical conditions. Kappa statistics were utilized to evaluate both intra‐ and inter‐observer agreement (Landis & Koch, 1977).

AP cases were diagnosed based on the identification of at least one tooth with periapical radiolucency outpacing twice the width of the periodontal ligament space (Bornstein et al., 2011; Low et al., 2008) and having PAI >2 (Costa et al., 2014). Individuals without clinical and radiographic evidence of AP with PAI ≤2 were excluded from the study.

### Statistical analysis

#### Sample size calculation

The sample size calculation was based on the null hypothesis that the prevalence of periodontitis in the present sample was the same as reported in a previous study (Tibúrcio‐Machado et al., 2021). It was assumed that the study cohort would have a 10% higher prevalence. With significance (*α*) set at 0.05 and statistical power (*β*) at 0.80, the calculated sample size was 110 subjects. Given a nonresponse rate of 20%, 149 participants were planned for inclusion, ensuring adequate statistical power for the study.

#### Descriptive and inferential statistics

Statistical analysis was conducted using ad hoc software (STATA BE, version 17, StataCorp LP, Texas, United States), and the level of significance was set a priori at 5%. Continuous variables were expressed as Mean with a 95% Confidence Interval (CI); categorical data were tabulated as the number of observations (percentage‐%). After the Shapiro–Wilk test for verification of data distribution, Kruskal–Wallis and Chi‐squared tests were used to compare patients' characteristics according to the categories of adherence to MD, level of physical activity, perceived stress and sleep quality. These lifestyle factors were categorized as binary variables.

#### Logistic regression models

A multivariable logistic regression model was conducted to evaluate the association between lifestyle patterns (independent variable) and the occurrence of AP cases (dependent variable). The association between AP and lifestyle choices was expressed as crude and adjusted odds ratios (ORs). ORs were adjusted for age, gender, smoking, diabetes, AR, IBDs, osteoporosis and DMFT, which are parameters that could affect AP phenotype and were selected according to external knowledge. The Chunk Test was then performed to select the variables with higher predictive value. The model was chosen based on the highest area under the curve (AUC) that offers the greatest ability to discriminate the model between those who have AP and those who do not, based on the lowest Aikakes (AIC) and Bayesian (BIC) Information Criterion, to balance the reduced number of variables. Smoking was included as a predictor in the final model.

## RESULTS

### Participant characteristics

**FIGURE 1:**
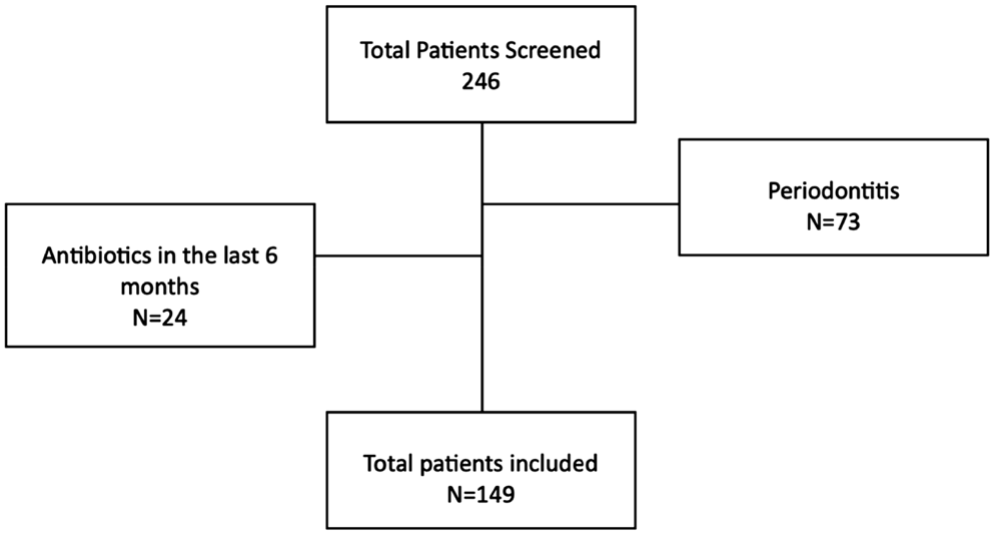
Flowchart for patients' selection.

A total of 149 patients were enrolled in the study after informed consent acceptance (Figure 1). The overall characteristics of the study population are shown in Table 1. All individuals evaluated for eligibility, decided to participate, were included in the study, and took part in the analysis. The mean age of the study population was 52.03 ± 16.43 years, and most of the included patients were females (56.38%) and never smokers (45.64%). Over half of the population had periapical lesions (56.38%). Sleep quality is considered insufficient in 41% of the population and physical activity level in 49%. Furthermore, adherence to the MD is high in 53.71% of patients, whilst almost 70% reported a low level of perceived stress.

**TABLE 1:** Patient's characteristics.

Variables	Mean (standard deviation) number (percentage)
Age	52.03 ± 16.43
Gender	
Male	65 (43.62%)
Female	84 (56.38%)
Occupation	
Unemployed	38 (25.50%)
Employed	70 (46.98%)
Retired	41 (27.52%)
Education	
Elementary school	48 (32.21%)
High school	68 (45.64%)
University	33 (22.15%)
Smoking	
Nonsmoker	68 (45.64%)
Ex‐smoker	44 (29.53%)
Smoker	37 (24.83%)
Diabetes	
No	122 (81.88%)
Familiarity	22 (14.77%)
Yes	5 (3.36%)
Rheumatoid arthritis	
No	143 (95.57%)
Familiarity	3 (2.01%)
Yes	3 (2.01%)
Inflammatory bowel diseases (IBDs)	
No	146 (97.99%)
Familiarity	2 (1.34%)
Yes	1 (0.67%)
Osteoporosis	
No	133 (89.26%)
Familiarity	6 (4.03%)
Yes	10 (6.71%)
Brushing	
Never	2 (1.34%)
Occasionally	40 (26.85%)
2 or more/day	107 (71.81%)
Interproximal hygiene	
No	59 (39.60%)
Floss	45 (30.20%)
Interproximal toothbrushes	32 (21.48%)
Floss + interproximal toothbrush	13 (8.72%)
Level of physical activity	
Low	65 (43.62%)
High	84 (56.38%)
Apical periodontitis	
No	65 (43.62%)
Yes	84 (56.38%)
Sleep quality	
Low	72 (41.14%)
High	103 (58.86%)
Perceived stress	
Low	122 (69.71%)
High	53 (30.29%)
Mediterranean diet adherence	
Low	81 (53.71%)
High	94 (46.29%)
Level of physical activity	
Moderate/low	86 (49.14%)
High	89 (50.86%)

*Note*: Results of continuous variables are reported as mean [95% confidence interval] and results of binary and categorical variables are expressed as the number of observations (proportion).

Abbreviations: aMed, alternate Mediterranean diet score; AR, rheumatoid arthritis; IBDs, inflammatory bowel diseases; *N* (%), number of observation (proportion); PAI score, periapical index score; SD, standard deviation.

The available sociodemographic variables are compared for high/low adherence to the MD, high/low‐moderate level of physical activity (Table 2), high/low stress and high/low quality of sleep (Table 3) to evaluate the presence of differences in these groups regarding the DMFT score and the presence of AP.

**TABLE 2:** Characteristics of patients categorized by adherence to the Mediterranean diet (aMed Score) and physical activity.

Variable	aMed		*p*‐value^a^	Level of physical activity		*p*‐value^a^
	Low (0–4)	High (5–9)		Low/moderate	High	
	*n* = 81	*n* = 68		*n* = 86	*n* = 63	
Age	50.73 ± 17.10	53.56 ± 15.59	.51	53.15 ± 15.88	50.49 ± 17.17	.46
Gender						
Male	40 (49.38%)	25 (36.76%)	.13	34 (40.48%)	31 (47.69%)	.40
Female	41 (50.62%)	43 (63.24%)		50 (59.52%)	34 (52.31%)	
Occupation						
Employed	23 (28.40%)	15 (22.06%)	.54	26 (30.95%)	12 (18.46%)	.21
Unemployed	36 (44.44%)	34 (50.00%)		36 (42.86%)	34 (52.31%)	
Retired	22 (27.16%)	19 (27.94%)		22 (26.19%)	19 (29.23%)	
Education						
Elementary school	23 (28.40%)	25 (36.76%)	.62	27 (32.14%)	21 (32.31%)	1.00
High school	38 (46.91%)	30 (44.12%)		38 (45.24%)	30 (46.15%)	
University	20 (24.69%)	13 (19.12%)		19 (22.62%)	14 (21.54%)	
Smoking						
Nonsmoker	36 (44.44%)	32 (47.06%)	.69	70 (41.67%)	37 (55.22%)	.18
Ex‐smoker	23 (28.40%)	21 (30.88%)		51 (30.36%)	16 (23.88%)	
Smoker	22 (27.16%)	15 (22.06%)		47 (27.98%)	14 (20.90%)	
Diabetes						
No	69 (85.19%)	53 (77.94%)		42 (50.00%)	26 (40.00%)	
Familiarity	9 (11.11%)	13 (19.12%)	.18	23 (27.38%)	21 (32.31%)	.93
Yes	3 (3.70%)	2 (2.94%)		19 (22.62%)	18 (27.69%)	
RA						
No	80 (98.77%)	63 (92.65%)		82 (97.62%)	61 (93.85%)	
Familiarity	1 (1.23%)	2 (2.94%)	.12	1 (1.19%)	2 (3.08%)	.55
Yes	0 (0%)	3 (4.41%)		1 (1.19%)	2 (3.08%)	
IBDs						
No	80 (98.77%)	66 (97.06%)	.72	83 (98.81%)	63 (96.92%)	.72
Familiarity	1 (1.23%)	1 (1.47%)		1 (1.19%)	1 (1.54%)	
Yes	0 (0%)	1 (1.47%)		0 (0%)	1 (1.54%)	
Osteoporosis						
No	75 (92.59%)	58 (85.29%)		77 (91.67%)	56 (86.15%)	
Familiarity	2 (2.47%)	4 (5.88%)	.36	2 (2.38%)	4 (6.15%)	.47
Yes	4 (4.94%)	6 (8.82%)		5 (5.95%)	5 (7.69%)	
Brushing						
Never	2 (2.47%)	0 (0%)		1 (1.19%)	1 (1.54%)	
Occasionally	27 (33.33%)	13 (19.12%)	. ***03*** *	25 (23.76%)	15 (23.08%)	.73
2 or more times/day	52 (64.20%)	55 (80.88%)		58 (69.05%)	49 (75.38%)	
Interproximal higiene						
No	39 (48.15%)	20 (29.41%)		35 (41.67%)	24 (36.92%)	
Floss	19 (23.46%)	26 (38.24%)	.09	24 (28.57%)	21 (32.31%)	.63
Apical periodontitis						
No	18 (22.22%)	47 (69.12%)		17 (20.24%)	51 (78.46%)	
Yes	63 (77.78%)	21 (30.88%)	. ***00*** ***	67 (79.76%)	14 (21.54%)	. ***00*** ***
PAI score	2.68 ± 1.59	0.00 ± 0.75	. ***00*** ***	2.32 ± 1.71	1.34 ± 1.73	. ***00*** ***
Decayed teeth (DT)	2.82 ± 2.67	3.38 ± 2.94	.44	2.90 ± 2.74	3.31 ± 2.88	.51
Missing teeth (MT)	4.25 ± 4.20	4.52 ± 4.67	.63	4.36 ± 4.07	4.41 ± 4.87	.71
Filled teeth (FT)	7.03 ± 4.27	7.54 ± 3.75	.44	7.88 ± 4.07	6.42 ± 3.87	. ***01*** *
DMFT	13.71 ± 6.96	15.41 ± 5.54	.27	14.80 ± 6.88	14.06 ± 5.66	.*31*
Sleep quality						
Low	40 (49.38%)	32 (34.04%)	. ***04*** *	50 (59.52%)	40 (43.96%)	1.00
High	41 (50.62%)	62 (65.96%)		34 (40.48%)	51 (56.04%)	
Perceived stress						
Low	58 (71.60%)	64 (68.09%)	.62	65 (77.38%)	57 (62.64%)	. ***02*** *
High	23 (28.40%)	30 (31.91%)		19 (22.62%)	34 (37.36%)	
Level of physical activity						
Mild/moderate	57 (70.37%)	29 (30.85%)	. ***00*** ***			
High	24 (29.63%)	65 (69.15%)				
Adherence to Mediterranean diet						
Low				62 (73.81%)	19 (20.88%)	. 00***
High				22 (26.19%)	72 (79.12%)	

*Note*: Results of continuous variables are reported as mean [95% confidence interval] and results of binary and categorical variables are expressed as numbers (proportion). Significance levels for estimates in bold and italics.

Abbreviations: aMed, alternate Mediterranean diet score; IBDs, Inflammatory Bowel Diseases; RA, Rheumatoid Arthritis; PAI score, Periapical index score.

^a^ *p*‐value of Mann–Whitney *U* test.

* *p* < .05.

** *p* < .01.

*** *p* < .001.

**TABLE 3:** Characteristics of patients categorized by sleep quality and perceived stress.

Variable	Sleep quality		*p*‐value^a^	Perceived stress		*p*‐value^a^
	Low (11–21)	High (0–10)		Low	High	
	*n* = 46	*n* = 103		*n* = 122	*n* = 27	
Age	53.56 ± 18.20	51.34 ± 15.62	.87	51.30 ± 16.00	55.29 ± 18.23	.15
Gender						
Male	27 (42.19%)	38 (44.71%)	.12	49 (40.16%)	16 (59.26%)	.08
Female	37 (57.81%)	47 (55.29%)		73 (59.84%)	11 (40.74%)	
Occupation						
Employed	22 (34.38%)	16 (18.82%)	.49	33 (27.05%)	5 (18.52%)	0.11
Unemployed	23 (35.94%)	47 (55.29%)		60 (49.18%)	10 (37.04%)	
Retired	19 (29.69%)	22 (25.88%)		29 (23.77%)	12 (44.44%)	
Education						
Elementary school	21 (32.81%)	27 (31.76%)	.56	38 (31.15%)	10 (37.04%)	.65
High school	31 (48.44%)	37 (43.53%)		55 (45.08%)	13 (48.15%)	
University	12 (18.75%)	21 (24.71%)		29 (23.77%)	4 (14.81%)	
Smoking						
Nonsmoker	22 (34.38%)	46 (54.12%)	.79	57 (46.72%)	11 (40.74%)	.51
Ex‐smoker	22 (34.38%)	22 (25.88%)		37 (30.33%)	7 (25.93%)	
Smoker	20 (31.25%)	17 (20.00%)		28 (22.95%)	9 (33.33%)	
Diabetes						
No	51 (79.69%)	71 (85.53%)		99 (81.15%)	23 (85.19%)	
Familiarity	10 (15.62%)	12 (14.12%)	.28	19 (15.57%)	3 (11.11%)	.90
Yes	3 (4.69%)	2 (2.35%)		4 (3.28%)	1 (3.70%)	
AR						
No	80 (98.77%)	81 (95.29%)		117 (95.90%)	26 (96.30%)	
Familiarity	1 (1.23%)	2 (2.35%)	.21	3 (2.46%)	0 (0.00%)	.70
IBDs						
No	80 (98.77%)	85 (100.0%)	.73	121 (99.18%)	25 (92.59%)	.08
Familiarity	1 (1.23%)	0 (0.0%)		1 (0.82%)	1 (3.70%)	
Yes	0 (0%)	0 (0.0%)		0 (0%)	1 (3.70%)	
Osteoporosis						
No	57 (89.06%)	76 (89.41%)		109 (89.34%)	24 (88.89%)	
Familiarity	2 (3.12%)	4 (4.71%)	.34	5 (4.10%)	1 (3.70%)	*1.00*
Yes	5 (7.81%)	5 (5.88%)		8 (6.56%)	2 (7.41%)	
Brushing						
Never	0 (0.0%)	2 (2.35%)		2 (1.64%)	0 (0.00%)	
Occasionally	19 (29.69%)	21 (24.71%)	. ***03*** *	31 (25.41%)	9 (33.33%)	.*64*
2 or more/day	45 (70.31%)	62 (72.94%)		89 (72.95%)	18 (66.67%)	
Interproximal hygiene						
No	28 (43.75%)	31 (36.47%)		48 (39.34%)	11 (40.74%)	
Floss	19 (29.69%)	26 (30.59%)	.12	39 (31.97%)	6 (22.22%)	.*06*
Interproximal toothbrush	11 (17.19%)	21 (24.71%)		28 (22.95%)	4 (14.81%)	
Floss+ interproximal toothbrush	6 (9.38%)	7 (8.24%)		7 (5.74%)	6 (22.22%)	
Apical periodontitis						
No	7 (10.94%)	61 (71.76%)		62 (50.82%)	3 (11.11%)	
Yes	57 (89.06%)	24 (28.24%)	. ***00*** ***	60 (49.18%)	24 (88.89%)	. ***00*** ***
PAI score	3.33 ± 1.12	1.27 ± 1.65		1.62 ± 1.74	3.18 ± 1.34	
Decayed teeth (DT)	3.43 ± 3.39	2.92 ± 2.49	.88	3.22 ± 2.83	2.40 ± 2.59	.10
Missing teeth (MT)	5.15 ± 4.25	4.03 ± 4.45	.11	4.09 ± 4.06	5.70 ± 5.62	.22
Filled teeth (FT)	7.79 ± 4.43	7.05 ± 3.85	.97	7.36 ± 4.08	6.81 ± 3.87	.67
DMFT	16.30 ± 6.88	13.67 ± 6.01	.15	14.36 ± 6.45	15.03 ± 6.16	.85
Sleep quality						
Low				50 (59.52%)	40 (43.96%)	. ***00*** ***
High				34 (40.48%)	51 (56.04%)	
Perceived stress						
Low	58 (71.60%)	64 (68.09%)	. ***03*** *			
High	23 (28.40%)	30 (31.91%)				
Livel of physical activity						
Mild/moderate	50 (55.56%)	34 (40.00%)	.*43*	65 (53.28%)	19 (35.85%)	. ***02*** *
High	40 (44.44%)	51 (60.00%)		57 (46.72%)	34 (64.15%)	
Adherence to Mediterranean diet						
Low	53 (58.89%)	28 (32.94%)	. ***00*** ***	58 (47.54%)	23 (43.40%)	. ***00*** ***
High	37 (41.11%)	57 (67.06%)		64 (52.46%)	30 (56.60%)	

*Note*: Results of continuous variables are reported as mean [95% confidence interval] and results of binary and categorical variables are expressed as numbers (proportion). Significance levels for estimates in bold and italics.

Abbreviations: aMed, alternate Mediterranean diet score; IBDs, Inflammatory Bowel Diseases; AR, Rheumatoid Arthritis; PAI score, Periapical index score.

^a^ *p*‐value of Mann–Whitney *U* test.

* *p* < .05.

** *p* < .01.

*** *p* < .001.

For each questionnaire, the diagnostic accuracy for the discrimination of AP was calculated (diagnostic accuracy calculated as the area under the ROC curve). Statistically significant differences for AP were found in subjects with low/high adherence to the MD (*p* = .00), low/high physical activity (*p* = .00), low/high sleep quality (*p* = .00) and low/high stress (*p* = .00). No statistically significant differences were traced between groups for the DMFT score variable.

### Outcome data

#### Intra‐ and inter‐observer agreement

The intra‐examiner agreement for the PAI score was kappa = 0.77 (95% CI: 0.67–0.8; *p* < .05) for the first examiner and kappa = 0.79 (95% CI: 0.72–0.85; *p* < .05) for the second examiner; inter‐examiner agreement resulted to be substantial with kappa = 0.75 (95% CI: 0.68–0.82; *p* < .05).

#### Adherence to MD

High adherence to MD was significantly associated with a lower prevalence of AP (30.88%) compared to those with low adherence (69.12%) (*p* = .00). DMFT did not report statistically significant differences between high/low adherence to MD (Table 2). The combination of high aMed scores and the habitual intake of certain MD foods resulted in lower odds of AP occurrence, even after adjustment (OR = 3.68 [1.24–10.83] *p* = .01). Furthermore, a higher PAI score is significantly associated with lower MD adherence. Accordingly, the mean PAI score was 0.00 ± 0.75 for patients with high MD adherence, whilst for low MD adherence was 2.68 ± 1.59 (*p* = .00).

When comparing the adherence to MD with sleep quality and the level of physical activity, it emerged that, respectively, 65.96% and 69.15% of patients reporting high adherence to MD also reported high sleep quality. On the contrary, 71.60% of patients reporting low adherence to MD also reported low sleep quality, and 70.37% reported low/moderate physical activity levels. These results demonstrate statistically significant differences in terms of adherence to MD between the high/low sleep quality group (*p* = .04) and the low‐moderate/high physical activity group (*p* = .00). On the contrary, no significant differences are highlighted as to perceived stress.

#### Physical activity

Sixty‐three participants reached a high physical activity level, whilst the other 86 were in the low/moderate physical activity level category (Table 2). A high level of physical activity was significantly associated with a lower prevalence of AP (21.54%) compared to a low/moderate physical activity level (79.76%). Additionally, the PAI score is significantly associated with a lower level of physical activity. Active patients reported a mean PAI of 1.34 ± 1.73; on the contrary, inactive patients had a mean PAI of 2.32 ± 1.71 (*p* = .00). However, DMFT did not report statistically significant differences between the two groups. Amongst the patients displaying high levels of physical activity, 56.04% also reported high sleep quality and only 43.96% had low sleep quality. However, no significant association was demonstrated between physical activity and sleep quality.

#### Sleep quality

Poor sleep quality presented a significantly higher incidence of AP than patients reporting high sleep quality (*p* = .03). Precisely 57 patients (89.06%) reporting poor sleep quality exhibited periapical lesions; on the contrary, only 24 patients (28.24%) reporting high sleep quality had AP. Furthermore, PAI was significantly worse in subjects with poor sleep quality compared to good sleep quality (*p* = .00). On the contrary, DMIT did not show any significant change amongst the two groups (*p* = .15). It was also observed that patients with a higher sleep quality tend to brush more often during the day compared to those patients reporting low sleep quality (*p* = .03). Moreover, 68.09% and 67.06% of patients reporting high sleep quality report low perceived stress (*p* = .03) and high adherence to MD (*p* = .00).

#### Perceived stress

Participants with high perceived stress displayed a significantly higher incidence of AP (88.89%) compared to those having low perceived stress (49%) (*p* = .00). Additionally, the PAI score was significantly worse in patients who reported a high level of stress compared to nonstressed patients [3.18 ± 1.34 vs. 1.62 ± 1.74 (*p* = .00), respectively]. However, DMFT did not report statistically significant differences between high and low perceived stress groups (*p* = .85). Additionally, patients classified as highly stressed, 56.04%, also show significantly higher scores on the PSQI questionnaire (*p* = .00).

#### Regression model

The final regression model is shown in Table 4. The proposed model is statistically significant (*p* = .00) with a pseudo *R* ^2^ of around 32%. The best model selected has AUC = 0.75, AIC = 185.0 and BIC = 199.9. The model shows that poor MD adherence, poor sleep quality and increased DMFT score are statistically significant predictors of AP. Poor adherence to MD increases the probability of incurring AP by about 4 times (OR = 3.68; confidence interval [CI]: 1.24–10.83; *p* = .01).

**TABLE 4:** Multivariable logistic regression analysis for prediction of AP by measures of adherence to Mediterranean diet (aMed score), sleep quality (PSQI), smoking and DMFT.

Best model (AUC = 0.75; AIC = 185.0; BIC = 199.9)		
LR *χ* ^2^	Prob >*χ* ^2^	Pseudo *R* ^2^
28.63	.00	.3194

Presence of AP	OR	SE	*z*	*p*‐value	95% CI	
					Lower	Greater
aMed	3.68	2.02	2.36	. ***01*** *	1.24	10.83
PSQI	3.04	1.18	2.87	. ***00*** ***	1.42	6.50
Smoking	2.26	.28	1.01	.31	.80	1.97
DMFT	1.07	.*03*	2.32	. ***02*** *	1.01	1.14
_cons	.14	.09	−3.13	. 00***	.04	.48

*Note*: Significance levels for estimates are in bold and italics.

Abbreviations: AIC, Akaike information criterion; aMed, alternate Mediterranean diet score; AUC, area under the curve; BIC, Bayesian information criterion; CI, confidence interval; DMFT, decayed, missing and filled teeth; LR, likelihood ratio; PSQI, Pittsburgh Sleep Quality Index.

* *p* < .05.

** *p* < .01.

*** *p* < .001.

Furthermore, having poor sleep quality increased the odds of having AP by about 3 times (OR = 3.04; CI: 1.42–6.50; *p* = .00). On the contrary, smoking increased the probability of AP by 2.26 times, but the results were not statistically significant (OR = 2.26; CI: 0.80–1.97; *p* = .31). Finally, as the DMFT score increases, there is a statistically significant higher likelihood of experiencing AP (OR = 1.07; CI: 1.01–1.14; *p* = .02).

## DISCUSSION

Results from the present cross‐sectional study show that poor adherence to MD, reduced physical activity, high perceived stress and inadequate sleep quality are significantly associated with a higher incidence of AP and increased PAI scores. Regression analysis revealed that poor adherence to MD increased the odds of developing AP by four times, whilst inadequate sleep quality increased the odds by three times. These findings support the correlation between an unhealthy lifestyle and more extensive PAI, as evidenced by higher PAI scores in individuals reporting unhealthy lifestyle patterns. Furthermore, each one‐point increase in the DMFT index was associated with an increased probability of developing AP, confirming caries as a significant factor in AP onset (Duncan et al., 2019). Notably, the significance of these findings is not attributed to factors such as plaque control, as they remained similar across all categories.

To the best of our knowledge, this is the first clinical study formulating the hypothesis of a significant association between MD adherence, sleep quality, physical activity level and perceived stress (exposure) with the presence and severity of AP (outcomes). Overall, only half of the patients in the present cohort reported high values of physical activity (56.38%). Physical activity was assessed by the IPAQ which is the most widely used physical activity questionnaire (Lee et al., 2011). The overall score estimates metabolic expenditure and was designed to categorize people into low (>700 MET), moderate (700 ≤ × ≤ 2519 MET) or high activity (<2519 MET). Precisely weekly activity times were calculated from days and minutes of vigorous, moderate and walking/light activity. According to the current findings, physical inactivity (>700 MET) was associated with a higher prevalence of AP and higher PAI scores. Indeed, only 21.54% of physically active patients developed AP; on the contrary, almost 80% of inactive patients were diagnosed with periapical lesions. To date, no data correlate physical inactivity to a higher incidence of AP. However, previous studies have demonstrated an association between low exercise and periodontitis (Marruganti et al., 2022). Despite differences in aetiology and pathogenesis, periodontitis and AP are both polymicrobial infections sharing a common microbiota and are characterized by an increased systemic level of cytokines (Cotti et al., 2011; Sundqvist, 1992). Therefore, it could be plausible to hypothesize that a sedentary lifestyle may also have a role in the development of AP, as highlighted by our findings. Furthermore, it was demonstrated that regular physical exercise has an anti‐inflammatory effect and can control pro‐inflammatory cytokines playing a role in the etiopathogenesis of AP (Conti et al., 2020; Metsios et al., 2020).

Our results reveal a significant association between low adherence to MD and increased incidence of periapical lesions. The study utilized the QueMD questionnaire to assess patients' adherence to MD and calculated the Alternate MD score (aMed) based on the questionnaire responses. Participants reporting food consumption levels above the Italian National guidelines received 1 point for each item typical of the MD. The aMed score was calculated and then dichotomized into low adherence (aMed <5) and high adherence (aMed >4) groups. Notably, 77.78% of study subjects who reported low adherence to MD exhibited endodontic lesions. This aligns with prior animal studies that have demonstrated that a hyperlipidemic diet could increase the progression and severity of AP (Brasil et al., 2021; Xiao et al., 2023). Despite evidence from animal studies, no clinical study has evaluated the association between diet and the presence and extent of periapical lesions. However, it was recently demonstrated that individuals who reported low MD adherence and lead a sedentary lifestyle have 10 times the odds of developing severe forms of periodontitis (Marruganti et al., 2022). Furthermore, from a biological standpoint, it is well‐known that the consumption of a Western‐style diet rich in processed foods induces a state of LGSI (Malesza et al., 2021). Our findings suggest that high MD adherence is inversely related to AP, supposedly due to the synergistic anti‐inflammatory potential of the single MD components.

The current observational study also indicated that high perceived stress and poor sleep quality are associated with AP and higher severity of PAI. To date, there is no evidence on this matter; however, few studies hypothesized the presence of an association between perceived stress and periodontitis (Coelho et al., 2020; Marruganti et al., 2023), as well as poor sleep quality and periodontitis (Marruganti et al., 2023). Furthermore, it was demonstrated that prolonged sleep deficiency can lead to chronic, LGSI and is associated with various diseases that have an inflammatory component (Besedovsky et al., 2019).

Precisely, chronic psychological stress activates the hypothalamic–pituitary axis excessively, resulting in elevated cortisol production and release. Similarly, it triggers the sympathetic nervous system, leading to effects comparable to those caused by poor sleep quality. This, in turn, contributes to an upsurge in markers of systemic inflammation, including C‐reactive proteins and interleukin‐6 (Hall, 2015; Marruganti et al., 2023; Nakata, 2012), whose negative influence on periapical health was demonstrated in a previous study (Conti et al., 2020).

No significant association was found between DMFT and lifestyle habits in the present study. This result contrasts with a previous investigation on 71 069 Japanese children which disclosed that late bedtime and short sleep duration were both consistently associated with increased risk of caries in deciduous teeth (Chen et al., 2018). This discrepancy with our findings may be attributed to the different study populations and the caries assessment methods used in both studies.

The decision to exclude patients who had taken antibiotics in the last 6 months was based on their anti‐inflammatory effects (Al‐Banna et al., 2013). Indeed, it was demonstrated that antibiotics influence the production of cytokines, chemotaxis and recruitment of leukocytes, production of reactive oxygen species, the process of phagocytosis and autophagy and apoptosis of leukocytes (Al‐Banna et al., 2013). Recent antibiotic use could hinge on the actual effect that good/bad lifestyle habits could have on the development of AP and its symptoms.

The odds ratios were adjusted to account for variables that could influence the outcome of the study. Whilst certain systemic conditions such as osteoporosis, diabetes, IBD and RA were included in the study, patients with CVD and cancer were not included, as these conditions are managed by a specialized unit within our department.

Nonetheless, this is the first clinical study formulating the hypothesis of a significant association between MD adherence, sleep quality, physical exercise and stress with AP and severity of periapical destruction. Lifestyle quality was assessed using reliable and validated questionnaires for the selected study population (Curcio et al., 2013; Gnagnarella et al., 2018; Mannocci et al., 2010; Mondo et al., 2021). Ultimately, the assessment of exposure (i.e., administration of questionnaires) and the outcome (i.e., clinical and radiographic examination) were performed by different operators blinded during outcome assessment, significantly improving the internal validity of the study.

In this study, we have primarily focused on the potential influence that lifestyle factors (i.e., MD, sleep quality, physical activity and stress) may have on the occurrence of AP and caries. However, it is known that oral health reflects various physiological, social and psychological attributes that play an important role in the overall quality of life (Hescot, 2017). Therefore, it is crucial to consider also the possibility that the presence of AP or other oral health issues may alter dietary choices, stress management, physical activity and sleep patterns, rather than the reverse. The relationship between dietary carbohydrates and dental‐systemic diseases exemplifies the intricate and potentially bidirectional nature of these associations (Hujoel, 2009). Similarly, our results may reflect a multifaceted interaction where lifestyle factors and oral health influence each other.

The present cross‐sectional study has some limitations. First, given the observational nature of the study, it is impossible to establish a longitudinal evaluation regarding the cause–effect relationship between the exposure and the outcome. Indeed, it can be employed only to build a hypothesis, and reverse causality cannot be excluded. Being a cross‐sectional study, data on lifestyle habits and AP status were collected simultaneously, without being able to establish temporality. Secondly, no molecular or immunological analysis was included in the study to support the biological plausibility of the hypothesized association, and no saliva or blood samples were collected, so assessments of inflammatory markers could not be performed. Thirdly, given the absence of studies regarding the effect of lifestyle on AP, the sample size calculation was performed based on the prevalence of AP. Fourthly, all the study subjects were prevalently of Caucasian ethnicity; thus, this homogeneity may limit the ability to detect variability in ethnicity‐related outcomes. Moreover, the risk of selection bias cannot be ruled out due to the study population being selected from patients coming to a Public Hospital. Furthermore, even though the major confounding factors were considered in the multiple models, the risk of residual confounding factors cannot be ruled out and no data were collected regarding the medications taken by each patient. Ultimately, despite most of the cases do not justify the patient's exposure to a cone‐beam computed tomography, it is recognized as the gold standard for AP detection (Cotti & Schirru, 2022). Indeed, periapical radiographs have a limited ability to detect changes in periapical bone and a high risk of underdiagnosis (An et al., 2016).

## CONCLUSION

Results from the present study suggest a potential association between low adherence to MD and reduced sleep quality with the development of AP. Individuals with low adherence to MD and inadequate sleep quality faced respectively 4‐fold and 3‐fold increased odds of developing periapical lesions. Further research is essential to elucidate the causal mechanisms underlying these associations and to determine whether lifestyle adjustments could improve endodontic success rate.

## AUTHOR CONTRIBUTIONS

**Carlo Gaeta:** supervision, Data curation, Investigation, Methodology, **Giulia Malvicini:** writing—original draft and editing, **Dominga Di Lascio:** conceptualization, methodology, data curation, **Marco Martignoni:** supervision, writing—review and editing, **Gabriele Ragucci:** data curation, investigation, **Simone Grandini:** data curation, methodology, **Crystal Marruganti:** formal analysis, investigation, methodology.

## FUNDING INFORMATION

No external funding, apart from the support of the authors' institution, was available for this study.

## CONFLICT OF INTEREST STATEMENT

The authors deny any conflict of interest related to this study.

## ETHICS STATEMENT

Approved by the University Hospital of Siena Ethics Committee (Siena, Italy), Area Vasta Toscana Sud Est, protocol number 18993/2021.

## PATIENT CONSENT STATEMENT

All enrolled patients were informed about the study protocol and were asked to read and sign the informed consent. The present study was conducted according to the declaration of Helsinki.

## Data Availability

The datasets used and/or analysed during the current study are available from the corresponding author upon reasonable request.

## References

[iej14165-bib-0045] Ørstavik, D. , Kerekes, K. & Eriksen, H.M. (1986) The periapical index: a scoring system for radiographic assessment of apical periodontitis. Dental Traumatology, 2, 20–34. Available from: 10.1111/J.1600-9657.1986.TB00119.X 3457698

[iej14165-bib-0001] Al‐Banna, N.A. , Pavlovic, D. , Gründling, M. , Zhou, J. , Kelly, M. , Whynot, S. et al. (2013) Impact of antibiotics on the microcirculation in local and systemic inflammation. Clinical Hemorheology and Microcirculation, 53, 155–169. Available from: 10.3233/CH-2012-1583 22975936

[iej14165-bib-0002] American Association of Endodontists . (2013) Endodontic diagnosis. https://www.aae.org

[iej14165-bib-0060] An, G.K. , Morse, D.E. , Kunin, M. , Goldberger, R.S. & Psoter, W.J. (2016) Association of radiographically diagnosed apical periodontitis and cardiovascular disease: a hospital records–based study. Journal of Endodontics, 42, 916–920. Available from: 10.1016/J.JOEN.2016.03.011 27091354

[iej14165-bib-0003] Bach‐Faig, A. , Berry, E.M. , Lairon, D. , Reguant, J. , Trichopoulou, A. , Dernini, S. et al. (2011) Mediterranean diet pyramid today. Science and cultural updates. Public Health Nutrition, 14, 2274–2284. Available from: 10.1017/S1368980011002515 22166184

[iej14165-bib-0004] Besedovsky, L. , Lange, T. & Haack, M. (2019) The sleep‐immune crosstalk in health and disease. Physiological Reviews, 99, 1325–1380. Available from: 10.1152/PHYSREV.00010.2018 30920354 PMC6689741

[iej14165-bib-0005] Biswas, B. , Saha, R. , Haldar, D. & Saha, I. (2019) Level of stress perception and predictors of higher stress perception among informal primary caregivers of eastern Indian people living with HIV/AIDS. International Journal of Community Medicine and Public Health, 6, 4374–4380. Available from: 10.18203/2394-6040.IJCMPH20194497

[iej14165-bib-0006] Bornstein, M.M. , Lauber, R. , Sendi, P. & Von Arx, T. (2011) Comparison of periapical radiography and limited cone‐beam computed tomography in mandibular molars for analysis of anatomical landmarks before apical surgery. Journal of Endodontia, 37, 151–157. Available from: 10.1016/J.JOEN.2010.11.014 21238794

[iej14165-bib-0007] Brasil, S.C. , Santos, R.M.M. , Fernandes, A. , Lima, R.S. , Costa, C.A.S. , Pinto, K.M.M.D.C. et al. (2021) Influence of a high‐fat diet in the progression of apical periodontitis. Journal of Endodontia, 47, 600–605. Available from: 10.1016/J.JOEN.2020.12.015 33387552

[iej14165-bib-0008] Braz‐Silva, P.H. , Bergamini, M.L. , Mardegan, A.P. , De Rosa, C.S. , Hasseus, B. & Jonasson, P. (2018) Inflammatory profile of chronic apical periodontitis: a literature review. Acta Odontologica Scandinavica, 77, 173–180. Available from: 10.1080/00016357.2018.1521005 30585523

[iej14165-bib-0009] Chauhan, N. , Mittal, S. , Tewari, S. , Sen, J. & Laller, K. (2019) Association of apical periodontitis with cardiovascular disease via noninvasive assessment of endothelial function and subclinical atherosclerosis. Journal of Endodontia, 45, 681–690. Available from: 10.1016/j.joen.2019.03.003 31030979

[iej14165-bib-0010] Chen, H. , Tanaka, S. , Arai, K. , Yoshida, S. & Kawakami, K. (2018) Insufficient sleep and incidence of dental caries in deciduous teeth among children in Japan: a population‐based cohort study. The Journal of Pediatrics, 198, 279–286.e5. Available from: 10.1016/J.JPEDS.2018.03.033 29709344

[iej14165-bib-0011] Chiuve, S.E. , Fung, T.T. , Rexrode, K.M. , Spiegelman, D. , Manson, J.A.E. , Stampfer, M.J. et al. (2011) Adherence to a low‐risk, healthy lifestyle and risk of sudden cardiac death among women. JAMA, 306, 62–69. Available from: 10.1001/JAMA.2011.907 21730242 PMC3210472

[iej14165-bib-0012] Coelho, J.M.F. , Miranda, S.S. , da Cruz, S.S. , Trindade, S.C. , Passos‐Soares, J.S. , Cerqueira, E.M.M. et al. (2020) Is there association between stress and periodontitis? Clinical Oral Investigations, 24, 2285–2294. Available from: 10.1007/S00784-019-03083-9 31654249

[iej14165-bib-0013] Cohen, S. , Kamarck, T. & Mermelstein, R. (1983) A global measure of perceived stress, source. Journal of Health and Social Behavior, 24, 385. 6668417

[iej14165-bib-0014] Conti, L.C. , Segura‐Egea, J.J. , Cardoso, C.B.M. , Benetti, F. , Azuma, M.M. , Oliveira, P.H.C. et al. (2020) Relationship between apical periodontitis and atherosclerosis in rats: lipid profile and histological study. International Endodontic Journal, 53, 1387–1397. Available from: 10.1111/iej.13350 32573791

[iej14165-bib-0059] Costa, T.H.R. , Neto, J.A.D.F. , De Oliveira, A.E.F. , Maia, M.D.F.L.E. & De Almeida, A.L. (2014) Association between chronic apical periodontitis and coronary artery disease. Journal of Endodontics, 40(2), 164–167. Available from: 10.1016/J.JOEN.2013.10.026 24461397

[iej14165-bib-0015] Cotti, E. , Dessì, C. , Piras, A. & Mercuro, G. (2011) Can a chronic dental infection be considered a cause of cardiovascular disease? A review of the literature. International Journal of Cardiology, 148, 4–10. Available from: 10.1016/J.IJCARD.2010.08.011 20851474

[iej14165-bib-0016] Cotti, E. & Schirru, E. (2022) Present status and future directions: imaging techniques for the detection of periapical lesions. International Endodontic Journal, 55, 1085–1099. Available from: 10.1111/IEJ.13828 36059089

[iej14165-bib-0017] Curcio, G. , Tempesta, D. , Scarlata, S. , Marzano, C. , Moroni, F. , Rossini, P.M. et al. (2013) Validity of the Italian version of the Pittsburgh sleep quality index (PSQI). Neurological Sciences, 34, 511–519. Available from: 10.1007/S10072-012-1085-Y 22526760

[iej14165-bib-0018] Duncan, H.F. , Galler, K.M. , Tomson, P.L. , Simon, S. , El‐Karim, I. , Kundzina, R. et al. (2019) European Society of Endodontology position statement: management of deep caries and the exposed pulp. International Endodontic Journal, 52, 923–934. Available from: 10.1111/IEJ.13080 30664240

[iej14165-bib-0019] Duncan, H.F. , Kirkevang, L.‐L. , Peters, O.A. , El‐Karim, I. , Krastl, G. , Del Fabbro, M. et al. (2023) Treatment of pulpal and apical disease: the European Society of Endodontology (ESE) S3‐level clinical practice guideline. International Endodontic Journal, 56(Suppl), 3–295. Available from: 10.1111/IEJ.13974 37772327

[iej14165-bib-0020] Esposito, K. , Marfella, R. , Ciotola, M. , Di Palo, C. , Giugliano, F. , Giugliano, G. et al. (2004) Effect of a mediterranean‐style diet on endothelial dysfunction and markers of vascular inflammation in the metabolic syndrome: a randomized trial. JAMA, 292, 1440–1446. Available from: 10.1001/JAMA.292.12.1440 15383514

[iej14165-bib-0021] Franciosi, G. , Fornich, S. , Di Matteo, C. , Malvicini, G. , Marruganti, C. , Grandini, S. et al. (2024) Environmental risk factors analysis in paediatric oral health: a cross‐sectional study. European Journal of Paediatric Dentistry, 1, 1. Available from: 10.23804/ejpd.2024.1987 38655744

[iej14165-bib-0022] Frodermann, V. , Rohde, D. , Courties, G. , Severe, N. , Schloss, M.J. , Amatullah, H. et al. (2019) Exercise reduces inflammatory cell production and cardiovascular inflammation via instruction of hematopoietic progenitor cells. Nature Medicine, 25, 1761–1771. Available from: 10.1038/S41591-019-0633-X PMC6858591 31700184

[iej14165-bib-0023] Georgiou, A.C. , Crielaard, W. , Armenis, I. , de Vries, R. & van der Waal, S.V. (2019) Apical periodontitis is associated with elevated concentrations of inflammatory mediators in peripheral blood: a systematic review and meta‐analysis. Journal of Endodontia, 45, 1279–1295.e3. Available from: 10.1016/J.JOEN.2019.07.017 31542282

[iej14165-bib-0024] Gnagnarella, P. , Dragà, D. , Misotti, A.M. , Sieri, S. , Spaggiari, L. , Cassano, E. et al. (2018) Validation of a short questionnaire to record adherence to the Mediterranean diet: an Italian experience. Nutrition, Metabolism, and Cardiovascular Diseases, 28, 1140–1147. Available from: 10.1016/J.NUMECD.2018.06.006 30077491

[iej14165-bib-0025] Gomes, M.S. , Blattner, T.C. , Sant'Ana Filho, M. , Grecca, F.S. , Hugo, F.N. , Fouad, A.F. et al. (2013) Can apical periodontitis modify systemic levels of inflammatory markers? A systematic review and meta‐analysis. Journal of Endodontia, 39, 1205–1217. Available from: 10.1016/J.JOEN.2013.06.014 24041380

[iej14165-bib-0026] Hall, M.H. (2015) Reciprocal associations between job strain and disturbed sleep—opportunities for sleep health. Sleep, 38, 1007–1008. Available from: 10.5665/SLEEP.4798 26085296 PMC4481009

[iej14165-bib-0027] Hescot, P. (2017) The new definition of Oral health and relationship between oral health and quality of life. The Chinese Journal of Dental Research, 20, 189–192. Available from: 10.3290/j.cjdr.a39217 29181455

[iej14165-bib-0028] Hujoel, P. (2009) Dietary carbohydrates and dental‐systemic diseases. Journal of Dental Research, 88, 490–502. Available from: 10.1177/0022034509337700 19587153

[iej14165-bib-0029] Huttunen, M. , Kämppi, A. , Soudunsaari, A. , Päkkilä, J. , Tjäderhane, L. , Laitala, M.L. et al. (2023) The association between dental caries and physical activity, physical fitness, and background factors among Finnish male conscripts. Odontology, 111, 192–200. Available from: 10.1007/s10266-022-00717-5 35612763 PMC9810556

[iej14165-bib-0030] Kushner, R.F. & Sorensen, K.W. (2013) Lifestyle medicine: the future of chronic disease management. Current Opinion in Endocrinology, Diabetes, and Obesity, 20, 389–395. Available from: 10.1097/01.MED.0000433056.76699.5D 23974765

[iej14165-bib-0031] Landis, J.R. & Koch, G.G. (1977) The measurement of observer agreement for categorical data. Biometrics, 33, 159. Available from: 10.2307/2529310 843571

[iej14165-bib-0032] Lee, P.H. , Macfarlane, D.J. , Lam, T.H. & Stewart, S.M. (2011) Validity of the international physical activity questionnaire short form (IPAQ‐SF): a systematic review. International Journal of Behavioral Nutrition and Physical Activity, 8, 115. Available from: 10.1186/1479-5868-8-115 22018588 PMC3214824

[iej14165-bib-0033] Li, C.X. , Leng, J. & Xiang, K. (2024) Association of lifestyle behaviors and oral health care needs: mediating effects of inflammatory markers. Preventive Medicine, 184, 108003. Available from: 10.1016/j.ypmed.2024.108003 38754737

[iej14165-bib-0034] Low, K.M.T. , Dula, K. , Bürgin, W. & von Arx, T. (2008) Comparison of periapical radiography and limited cone‐beam tomography in posterior maxillary teeth referred for apical surgery. Journal of Endodontia, 34, 557–562. Available from: 10.1016/J.JOEN.2008.02.022 18436034

[iej14165-bib-0035] Malesza, I.J. , Malesza, M. , Walkowiak, J. , Mussin, N. , Walkowiak, D. , Aringazina, R. et al. (2021) High‐fat, Western‐style diet, systemic inflammation, and gut microbiota: a narrative review. Cells, 10, 3164. Available from: 10.3390/CELLS10113164 34831387 PMC8619527

[iej14165-bib-0036] Malvicini, G. , Marruganti, C. , Leil, M.A. , Martignoni, M. , Pasqui, E. , de Donato, G. et al. (2024) Association between apical periodontitis and secondary outcomes of atherosclerotic cardiovascular disease: a case‐control study. International Endodontic Journal, 57, 281–296. Available from: 10.1111/IEJ.14018 38204179

[iej14165-bib-0037] Mannocci, A. , di Thiene, D. , del Cimmuto, A. , Masala, D. , Boccia, A. , de Vito, E. et al. (2010) International physical activity questionnaire: validation and assessment in an Italian sample. Italian Journal of Public Health, 7, 546. Available from: 10.2427/5694

[iej14165-bib-0038] Marrero, S.L. , Bloom, D.E. & Adashi, E.Y. (2012) Noncommunicable diseases: a global health crisis in a new world order. JAMA, 307, 2037–2038. Available from: 10.1001/JAMA.2012.3546 22665101

[iej14165-bib-0039] Marruganti, C. , Gaeta, C. , Romandini, M. , Ferrari Cagidiaco, E. , Parrini, S. , Discepoli, N. et al. (2023) Multiplicative effect of stress and poor sleep quality on periodontitis: a university‐based cross‐sectional study. Journal of Periodontology, 95, 125–134. Available from: 10.1002/JPER.23-0209 37477025

[iej14165-bib-0040] Marruganti, C. , Traversi, J. , Gaeta, C. , Ferrari Cagidiaco, E. , Parrini, S. , Discepoli, N. et al. (2022) Adherence to Mediterranean diet, physical activity level, and severity of periodontitis: results from a university‐based cross‐sectional study. Journal of Periodontology, 93, 1218–1232. Available from: 10.1002/JPER.21-0643 35119695 PMC9544461

[iej14165-bib-0041] Metsios, G.S. , Moe, R.H. & Kitas, G.D. (2020) Exercise and inflammation. Best Practice & Research. Clinical Rheumatology, 34, 101504. Available from: 10.1016/J.BERH.2020.101504 32249021

[iej14165-bib-0042] Mondo, M. , Sechi, C. & Cabras, C. (2021) Psychometric evaluation of three versions of the Italian perceived stress scale. Current Psychology, 40, 1884–1892. Available from: 10.1007/S12144-019-0132-8

[iej14165-bib-0043] Nakata, A. (2012) Psychosocial job stress and immunity: a systematic review. Methods in Molecular Biology, 934, 39–75. Available from: 10.1007/978-1-62703-071-7_3 22933140

[iej14165-bib-0058] National Research Institute for Food and Nutrition . (2003) Linee guida per una sana alimentazione Italiana. (Guidelines for an Italian Healthy Eating). National Research Institute for Food and Nutrition Published online. [Google Scholar].

[iej14165-bib-0044] Ng, Y.L. , Mann, V. & Gulabivala, K. (2011) A prospective study of the factors affecting outcomes of nonsurgical root canal treatment: part 1: periapical health. International Endodontic Journal, 44, 583–609. Available from: 10.1111/J.1365-2591.2011.01872.X 21366626

[iej14165-bib-0046] Page, R.C. & Eke, P.I. (2007) Case definitions for use in population‐based surveillance of periodontitis. Journal of Periodontology, 78, 1387–1399. Available from: 10.1902/JOP.2007.060264 17608611

[iej14165-bib-0047] Papapanou, P.N. , Sanz, M. , Buduneli, N. , Dietrich, T. , Feres, M. , Fine, D.H. et al. (2018) Periodontitis: consensus report of workgroup 2 of the 2017 world Workshop on the classification of periodontal and Peri‐implant diseases and conditions. Journal of Clinical Periodontology, 45(Suppl 20), S162–S170. Available from: 10.1111/JCPE.12946 29926490

[iej14165-bib-0048] Parkinson, M.D. , Stout, R. & Dysinger, W. (2023) Lifestyle medicine: prevention, treatment, and reversal of disease. The Medical Clinics of North America, 107, 1109–1120. Available from: 10.1016/j.mcna.2023.06.007 37806726

[iej14165-bib-0049] Ricci, C. , Schutte, A.E. , Schutte, R. , Smuts, C.M. & Pieters, M. (2020) Trends in alcohol consumption in relation to cause‐specific and all‐cause mortality in the United States: a report from the NHANES linked to the US mortality registry. The American Journal of Clinical Nutrition, 111, 580–589. Available from: 10.1093/AJCN/NQAA008 31978218

[iej14165-bib-0050] Siqueira, J.F., Jr. & Rôças, I.N. (2022) Present status and future directions: microbiology of endodontic infections. International Endodontic Journal, 55(Suppl 3), 512–530. Available from: 10.1111/iej.13677 34958494

[iej14165-bib-0051] Stringhini, S. , Sabia, S. , Shipley, M. , Brunner, E. , Nabi, H. , Kivimaki, M. et al. (2010) Association of socioeconomic position with health behaviors and mortality. JAMA, 303, 1159–1166. Available from: 10.1001/JAMA.2010.297 20332401 PMC2918905

[iej14165-bib-0052] Sundqvist, G. (1992) Ecology of the root canal flora. Journal of Endodontia, 18, 427–430. Available from: 10.1016/S0099-2399(06)80842-3 9796509

[iej14165-bib-0053] Tibúrcio‐Machado, C.S. , Michelon, C. , Zanatta, F.B. , Gomes, M.S. , Marin, J.A. & Bier, C.A. (2021) The global prevalence of apical periodontitis: a systematic review and meta‐analysis. International Endodontic Journal, 54, 712–735. Available from: 10.1111/iej.13467 33378579

[iej14165-bib-0054] Von Elm, E. , Altman, D.G. , Egger, M. , Pocock, S.J. , Gøtzsche, P.C. & Vandenbroucke, J.P. (2008) The strengthening the reporting of observational studies in epidemiology (STROBE) statement: guidelines for reporting observational studies. Journal of Clinical Epidemiology, 61, 344–349. Available from: 10.1016/J.JCLINEPI.2007.11.008 18313558

[iej14165-bib-0055] World Health Organization. World Health Organization . (2013) Oral health surveys: basic methods. Geneva: World Health Organization.

[iej14165-bib-0056] Xiao, S. , Lei, H. , Li, P. , Ma, D. , Chen, S. & Huang, X. (2023) Is oxidative stress involved in the hepatic inflammatory response to apical periodontitis? A comparative study in normal and hyperlipidaemic rat. International Endodontic Journal, 56, 722–733. Available from: 10.1111/IEJ.13907 36825367

[iej14165-bib-0057] Zhang, Y.B. , Pan, X.F. , Chen, J. , Cao, A. , Xia, L. , Zhang, Y. et al. (2021) Combined lifestyle factors, all‐cause mortality and cardiovascular disease: a systematic review and meta‐analysis of prospective cohort studies. Journal of Epidemiology and Community Health, 75, 92–99. Available from: 10.1136/jech-2020-214050 32892156

